# Diet and Immune Effects Trial (DIET)- a randomized, double-blinded dietary intervention study in patients with melanoma receiving immunotherapy

**DOI:** 10.1186/s12885-024-13234-1

**Published:** 2024-12-04

**Authors:** Rachel M. Farias, Yan Jiang, Erma J. Levy, Cindy Hwang, Jian Wang, Elizabeth M. Burton, Lorenzo Cohen, Nadim Ajami, Jennifer A. Wargo, Carrie R. Daniel, Jennifer L. McQuade

**Affiliations:** 1 Department of Melanoma Medical Oncology, The University of Texas MD Anderson Cancer Center, 1515 Holcombe, Unit 430, Houston, Texas 77030-4009 USA; 2 Department of Epidemiology, The University of Texas MD Anderson Cancer Center, Houston, TX USA; 3 Department of Biostatistics, The University of Texas MD Anderson Cancer Center, Houston, TX USA; 4 Department of Genomic Medicine, The University of Texas MD Anderson Cancer Center, Houston, TX USA; 5 Department of Integrative Medicine, The University of Texas MD Anderson Cancer Center, Houston, TX USA; 6 Department of Surgical Oncology, The University of Texas MD Anderson Cancer Center, Houston, TX USA

**Keywords:** Melanoma, Immunotherapy, Diet, Gut microbiome, Metabolome, Tumor immunity

## Abstract

**Background:**

Gut microbiome modulation is a promising strategy for enhancing the response to immune checkpoint blockade (ICB). Fecal microbiota transplant studies have shown positive signals of improved outcomes in both ICB-naïve and refractory melanoma patients; however, this strategy is challenging to scale. Diet is a key determinant of the gut microbiota, and we have previously shown that (a) habitual high dietary fiber intake is associated with an improved response to ICB and (b) fiber manipulation in mice impacts antitumor immunity. We recently demonstrated the feasibility of a controlled high-fiber dietary intervention (HFDI) conducted in melanoma survivors with excellent compliance and tolerance. Building on this, we are now conducting a phase II randomized trial of HFDI versus a healthy control diet in melanoma patients receiving ICB.

**Methods:**

This is a randomized, double-blind, fully controlled feeding study that will enroll 45 melanoma patients starting standard-of-care (SOC) ICB in three settings: adjuvant, neoadjuvant, and unresectable. Patients are randomized 2:1 to the HFDI (target fiber 50 g/day from whole foods) or healthy control diet (target fiber 20 g/day) stratified by BMI and cohort. All meals are prepared by the MD Anderson Bionutrition Core and are isocaloric and macronutrient-controlled. The intervention includes a 1-week equilibration period and then up to 11 weeks of diet intervention. Longitudinal blood, stool and tumor tissue (if available) are collected throughout the trial and at 12 weeks post intervention.

**Discussion:**

This DIET study is the first fully controlled feeding study among cancer patients who are actively receiving immunotherapy. The goal of the current study is to establish the effects of dietary intervention on the structure and function of the gut microbiome in patients with melanoma treated with SOC immunotherapies. The secondary endpoints include changes in systemic and tumor immunity, changes in the metabolic profile, quality of life, symptoms, disease response and immunotherapy toxicity.

**Trial registration:**

This protocol is registered with the U.S. National Institutes of Health trial registry, ClinicalTrials.gov, under the identifier NCT04645680. First posted 2020-11-27; last verified 2024-06.

## Background

Melanoma is the most aggressive and deadly form of common skin cancer. The introduction of immune checkpoint blockade (ICB) has dramatically improved the outcomes of patients with advanced melanoma. The US FDA has approved three different categories of ICB for melanoma: PD-1 inhibitors (nivolumab and pembrolizumab), CTLA-4 inhibitor (ipilimumab), and LAG-3 inhibitor (relatlimab) which considerably prolong the survival of patients with metastatic melanoma [1–3]. However, considerable heterogeneity in outcomes remains, with a high proportion of patients not responding to or developing resistance to ICB. There is an urgent need to identify predictors of response and develop new strategies to improve patient outcomes.

The microbes that reside in the human intestinal tract can significantly influence host metabolism, immunity, and disease. The microbiome plays a critical role in immune homeostasis, and dysbiosis has been found to contribute to the pathogenesis of multiple diseases, including obesity, diabetes, cardiovascular diseases, liver diseases, and autoimmune diseases, as well as carcinogenesis [4–8]. Recent studies from our group and others have demonstrated that the gut microbiome can also impact the response to immunotherapy and that microbiome modulation can impact the therapeutic response in preclinical models [9–11]. Fecal microbiota transplantation (FMT) of a favorable/pro-ICB-responsive microbiota into tumor-bearing mice can enhance the response to ICB [9, 11–13]. Small early-phase human studies (10–15 patients each) have further demonstrated that FMT can modulate the gut microbiome and overcome PD-1 resistance in some cancer patients [14, 15]. However, there are numerous challenges to the broad implementation of this strategy, particularly donor identification and retention, as well as the risk and availability of the procedure [16, 17]. 

Diet is a key determinant of the gut microbiome. Fiber-rich dietary patterns contain multiple microbiota-accessible carbohydrates, as well as other nutrients and bioactive compounds, with prebiotic properties to favorably impact the gut microbiome [9, 17–20]. In our observational cohort, we further found that patients reporting insufficient dietary fiber intake were less likely to respond to ICB [21, 22]. Dietary fiber deprivation in preclinical models similarly impacts immunity and the tumor response to anti-PD1 via the gut microbiome.

However, whether dietary modifications can translate to changes in the gut microbiome and immune response during immunotherapy in melanoma patients is still unknown. Our DIET trial (Effect of Diet on the Immune System in Patients with Stage III-IV Melanoma Receiving Immunotherapy, NCT04645680) is a controlled feeding study designed to assess the role of diet in modulating the gut microbiome in the context of ICB in patients with melanoma receiving SOC ICB. To our knowledge, this is the first controlled feeding study ever conducted in an actively ICB-treated cancer population.

## Methods/design

### Hypotheses and objectives

Given the effect of dietary fiber on the composition and function of the gut microbiome, we expect that HFDI in patients with melanoma receiving SOC ICB will modulate the structure and function of the microbiome and be safe and well tolerated. Our long-term hypothesis is that modulating the microbiome would impact systemic and antitumor immunity and improve the response to immunotherapy.

### Primary objective

The primary objective of the DIET study is to establish the effects of dietary intervention on the structure and function of the gut microbiome in patients with melanoma being treated with SOC ICB.

### Secondary objectives

The secondary objectives are to assess changes in systemic and tumor immunity, changes in metabolic profiles, the safety (adverse events) and tolerability (GI symptoms) of dietary interventions, and aspects of quality of life.

### Exploratory objectives

Exploratory objectives are to assess the associations of dietary interventions with disease outcomes (objective response rate [ORR] and progression-free survival [PFS] rates in unresectable cohorts and the recurrence rate [RR] in the adjuvant cohort) and explore predictors of response.

### Outcome measures

The primary outcome measure is longitudinal changes in the fecal microbiome profile. The secondary outcomes include changes in systemic and antitumor immunity, circulating blood metabolites derived from the host and microbiome, and clinical outcomes.

## Methods/design

### Overview of trial design

**Fig. 1:**
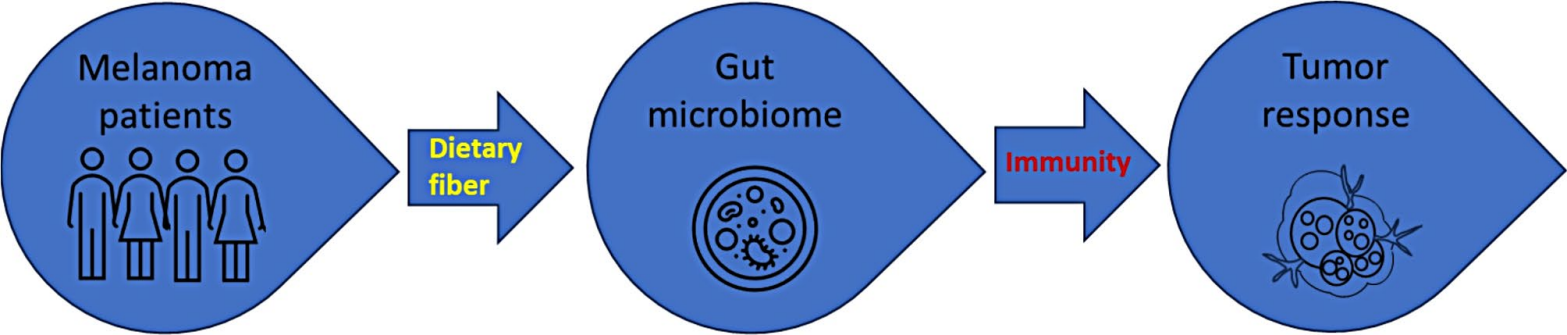
Conceptual of the study

The DIET study is a double-blind randomized controlled feeding trial (Fig. 1) of dietary intervention targeting the gut microbiome in patients with melanoma receiving standard-of-care anti-PD1 +/- anti-CTLA4 or anti-LAG3 immunotherapy. The target accrual is 45 patients with melanoma with planned initiation of SOC ICB in the neoadjuvant (*n* = 12), adjuvant (*n* = 21), or metastatic (*n* = 12) setting. The American Cancer Society (ACS) and American Institute for Cancer Research (AICR) have released dietary guidelines for cancer prevention, including increased intake of (1) fruits and vegetables, (2) legumes, and (3) whole grains and limiting consumption of (1) red and processed meat, (2) added sugars, and (3) alcohol [23, 24]. Both arms of the DIET study receive a diet that meets the ACS/AICR dietary recommendations for cancer prevention. However, in the control arm, dietary fiber intake is targeted to stay within the range consumed by the average melanoma patient and the US population (15–20 g daily) [25–27]. In the intervention arm, dietary fiber through whole foods is ramped up from a weekly average of 30 to up to 50 g per day.

Participants are randomized 2:1 to the HFDI or control diet, stratified by cohort and body mass index (< or ≥ 30). All meals, snacks, and energy-containing beverages are supplied during the study. The diets in both arms are isocaloric to meet each participants’ calculated energy (kcal) needs and are macronutrient-controlled (~ 20% protein, ~ 30% fat, ~ 50% carbohydrates). Individual menus are designed and monitored by a registered dietitian (RD) and prepared in the MD Anderson Bionutrition Research Core (BRC) kitchen.

Following a 1-week equilibration, during which all participants receive the control diet, the participants are provided with their assigned diet. Participants in the adjuvant and metastatic cohorts remain on the diet for 10 weeks, and participants in the neoadjuvant cohort remain on the diet until the day of their planned surgery.

### Recruitment and setting

**Fig. 2:**
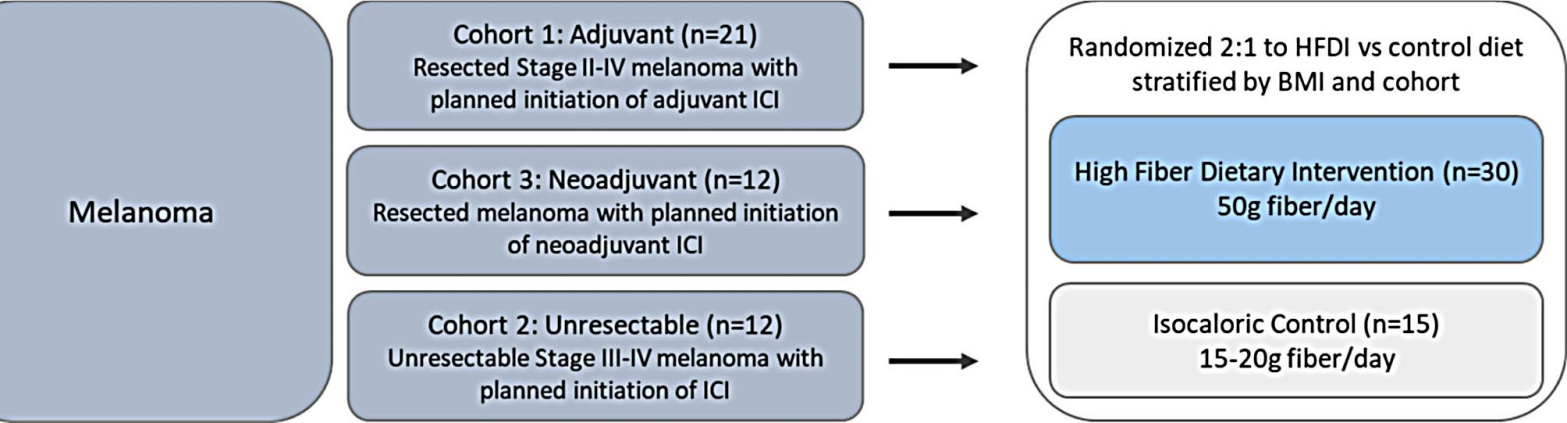
Study schema

The study is being conducted at The University of Texas MD Anderson Cancer Center (MDACC) Melanoma Clinic in Houston, Texas. Patients are identified into three cohorts (Fig. 2):

1. Stage III or IV melanoma with planned initiation of neoadjuvant ICB (*n* = 12).
2. Resected Stage II-IV melanoma patients with planned initiation of adjuvant ICB (*n* = 21).
3. Unresectable Stage III or IV melanoma with planned initiation of ICB and no prior ICB in the metastatic setting (*n* = 12).

Eligible patients are identified either by provider referral or by screening clinic schedules. Following the primary treating oncologist’s approval, potential patients are sent MyChart invitations with an overview of the study procedures. Interested patients are recruited during a clinic visit or via phone.

### Eligibility criteria

The full inclusion and exclusion criteria are included in Table 1.

**Table 1:** DIET eligibility criteria

Inclusion Criteria	Exclusion Criteria
• Age ≥ 18 years old. • Body Mass Index 18.5–40 kg/m2 • ECOG performance status of 0 or 1 • English-speaking • Self-reported willingness to exclusively eat the provided diets • Self-reported willingness to comply with scheduled visits, undergo venipuncture, and provide stool samples	• Ongoing colitis or diarrhea • History of ≥ Grade II colitis or diarrhea on ICB • Unresolved ≥ Grade III immune-related adverse event on ICB (other than endocrinopathy requiring hormone replacement) • History of active inflammatory bowel disease • Major gastrointestinal surgery (not including appendectomy or cholecystectomy) within 3 months of enrollment • History of total colectomy or bariatric surgery (bariatric surgery which does not disrupt the gastrointestinal lumen, i.e., restrictive procedures such as banding, are permitted). • Medical contraindications to intervention as determined by the treating physician • Self-reported major dietary restrictions related to intervention • Diagnosis of diabetes mellitus type I or type II that requires medical treatment or random glucose > 200 mg/dL • Antibiotic use within 21 days of planned start of equilibration diet • Condition requiring systemic treatment with either corticosteroids (> 10 mg daily prednisone equivalents) or other immunosuppressive medications within 14 days of study intervention administration. Inhaled or topical steroids, and adrenal replacement doses > 10 mg daily prednisone equivalents are permitted in the absence of active autoimmune disease. • Regularly taking probiotics, fiber supplements, or any other medication or supplement that could affect study outcome as determined by the principal investigator and unable/unwilling to discontinue for the purpose of the study. These agents must be discontinued at least 14 days prior to start of diet. • Currently consuming an average estimated daily fiber intake exceeding 20 g based on the results of the preliminary dietary assessment; vegetarian or vegan • Current smoker or heavy drinker (defined as > 14 drinks per week) or current self-reported illicit drug use • Uncontrolled concurrent illness or infection or psychiatric illness/social situations that would limit compliance with study requirements. • Plan for travel during the study that would preclude adherence to prescribed diets

### Informed consent

Potentially eligible participants are provided with a thorough review of the study by the study coordinator. Patients are informed in detail about the purpose, duration, assessments, and anticipated risks and benefits of the study. Written informed consent is obtained from all patients prior to screening. The consent document fulfills the requirements set forth by the Institutional Review Board (IRB) of MDACC. The protocol and all amendments are approved by the IRB.

## Study design and assessment (Table 2)

### Screening

Potential participants complete multiple food records and are interviewed by registered dietitians (RDs) to assess their usual or average baseline dietary fiber intake. The participants are asked to record everything they eat and drink for up to seven consecutive days. The estimated daily fiber is obtained by averaging fiber intake over three typical days. Participants with average fiber intake ≤ 20 g per day are eligible for the study.

**Table 2:**
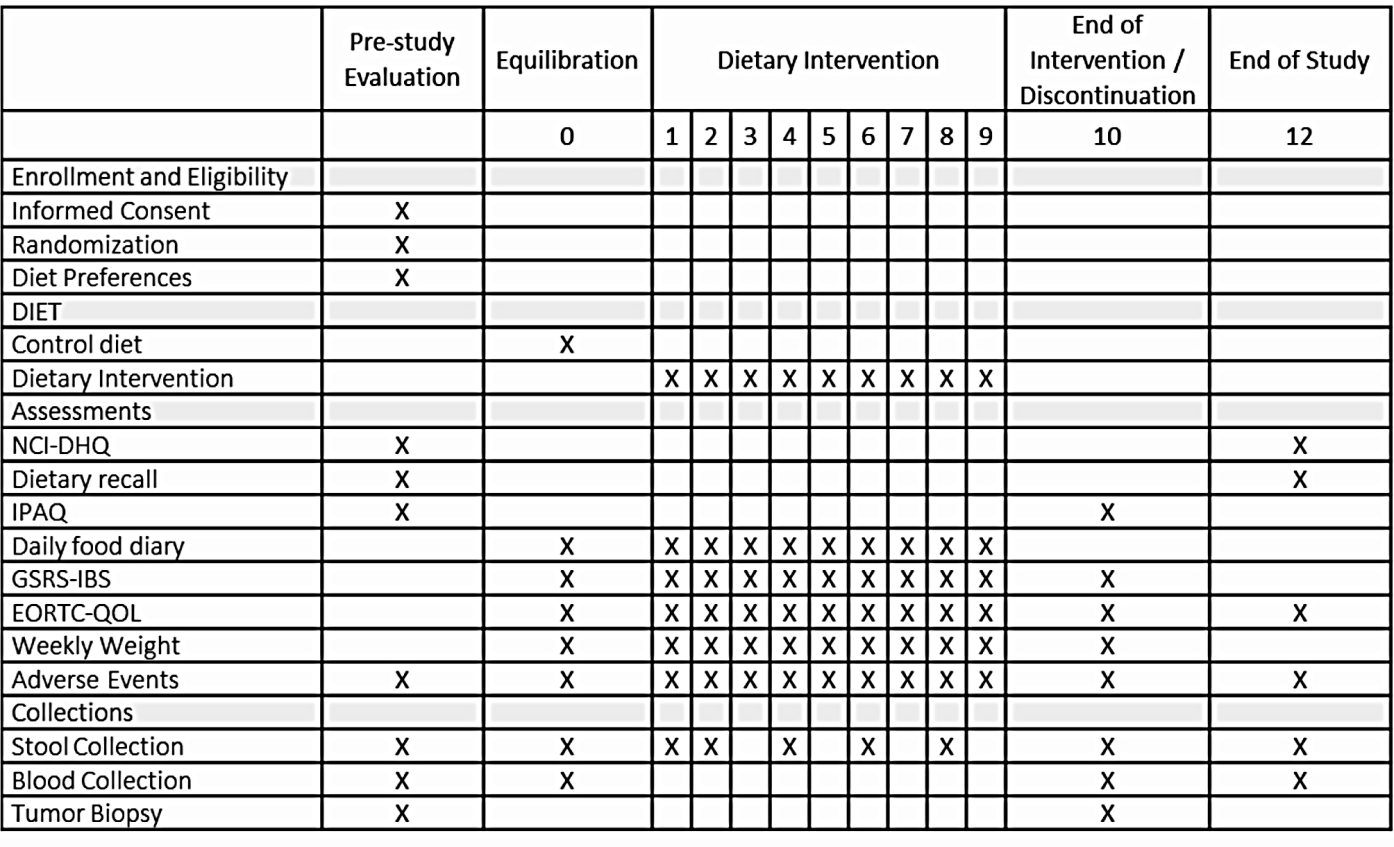
Study assessments

During screening, participants also complete a food preference questionnaire and the International Physical Activity Questionnaire (IPAQ), which are used to estimate energy needs [28] and design the participant’s diet. Stool samples, optional blood samples, and tumor tissue if required are collected.

Eligible participants who complete the screening assessments are randomized.

### Randomization

Eligible participants are randomized 2:1 to the HFDI or control arm stratified by BMI (< or ≥ 30) and the treatment cohort. The randomization is generated and allocated by the study biostatistician. The RD and study coordinator, who directly interacts with the participants during the study and the participants themselves, are blinded to diet allocation. The RD and data coordinator are not blinded and have access to the unblinded data.

### DIET intervention

The participants were provided with all meals, snacks, and energy-containing beverages for the duration of the study. The diets are isocaloric and individualized to each participants calculated energy needs. Menus are designed by an RD and prepared in the MDACC Bionutrition Research Core kitchen. Food may be either picked up from the MDACC or shipped in temperature-controlled packaging every 7 (± 4) days to participants. The participants are provided a scale to measure weekly weights and a Fitbit to monitor physical activity for the duration of the DIET intervention.

### Equilibration

All participants undergo a 1-week equilibration period during which they receive the control diet, which is a whole foods diet rich in fruits and vegetables, with a maximum of 18 oz. cooked red meat (including beef, pork, and lamb) per week, with little or no processed meats or added sugars that conform to AICR/ACS cancer prevention recommendations [23, 24]. Menus are based on participants’ caloric needs, with 30–35% of calories from fat, 45–55% from carbohydrates, 15–21% from protein, and a weekly average of 15–20 g of fiber/day, which reflects the average fiber intake of our melanoma population [25] and the US population based on observational data [26, 27]. 

### Dietary intervention

Starting at week 1, participants receive the HFDI or remain on the control diet. The HFDI is based on the ACS/AICR diet with the same macronutrient composition but with higher fiber content (example menu in Table 3). The HFDI starts at 30–35 g of fiber a day and is up titrated every 2 weeks to a maximum of 50 g of fiber a day based on tolerance. Participants may also be down titrated based on tolerance.

**Table 3:** Sample food menu

ACS/AICR Control Diet: 20 g Fiber menu (2,000 calories; Week averages: 1,972 calories, 20 g Fiber, 31% Fat, 49% CHO, 20% PRO)							
	Monday	Tuesday	Wednesday	Thursday	Friday	Saturday	Sunday
Breakfast	Ricotta Pancakes Apple Compote - (made with fresh apples) *Breakfast Turkey Sausage Fresh Pineapple Chunks	English Muffin Raspberry Chia Seed Jam (in-house)	Potato and *Turkey Sausage Hash White Toast	Oatmeal (oats) Strawberries	Breakfast Taco (*Turkey Breakfast Sausage, Eggs, Flour Tortilla), Roasted Tomato Salsa	Banana Muffins (wheat germ, whole wheat flour, Greek yogurt, banana) Apple	Bagel Cream Cheese Boiled Eggs Grapes
Lunch	Chop Salad - (romaine, tomato, carrots, chicken) Italian Dressing - (made in-house)	*Turkey Burger on white hamburger bun Tortilla Chips	White Pasta with *Turkey and Butternut Squash	Teriyaki Beef and Snap Peas White Rice	Mahi-mahi Spinach and White Rice Salad	Grilled Chicken Breast on white hamburger bun Tortilla Chips	*Turkey Patty Melt on white bread Baby Carrots
Dinner	White Penne Pasta with Pesto Sauce	Chicken Parmigiana	Chipotle Chicken Burrito Bowl with pinto beans and bell peppers	Honey Mustard Pork Tenderloin Herbed Corn and Orzo	White Spaghetti and *Turkey Meatballs	Beef Flank Steak White Rice Sesame Ginger Broccoli Pineapple Chunks	Baked Potato with Broccoli and Grilled Chicken
Snacks	Pretzels	Apple	Orange	Popcorn	Mango Smoothie (mangos, bananas, Greek yogurt)	Walnuts	Almonds
**High fiber diet: 50 g Fiber Menu (2,000 Calories; Week Averages: 2,046 Calories, 49 g Fiber, 32% Fat, 48% CHO, 20% PRO)**							
	**Monday**	**Tuesday**	**Wednesday**	**Thursday**	**Friday**	**Saturday**	**Sunday**
Breakfast	Banana Muffins (wheat germ, whole wheat flour, Greek yogurt, banana) *Turkey Breakfast Sausage Blueberries	Breakfast Black Bean Scramble, Whole Wheat Flour Tortillas Salsa Pear	Plain Greek Yogurt Granola (oats, chia seeds) Raspberries	Breakfast Sandwich(*turkey breakfast sausage, eggs, whole wheat bread) Orange	Whole Wheat Bagel Blueberry Compote Egg White Omelet Orange	Breakfast Quesadilla (*turkey breakfast sausage, eggs, whole wheat tortilla) Salsa	Whole Wheat Bagel Cream Cheese Feta Frittata
Lunch	Potato and Broccoli Hash with Chicken Edamame and Corn Succotash	Teriyaki Beef with Snap Peas and Brown Rice	*Turkey burger on whole wheat bun Jicama Fries Pineapple Chunks	Black Bean Chili Cornbread Muffin	Mahi-mahi Roasted Butternut Squash Saffron Quinoa	Shrimp Whole Wheat Pasta Salad (tomatoes, artichoke), walnuts)	Turkey Barley Soup(leeks, carrots, celery, barley, onions) Whole Wheat Crackers
Dinner	Beef Flank Whole Wheat Pasta with Garlic Basil Oil Sauteed Brussels Sprouts	Cilantro Lime Quinoa Bowl with Chicken (corn, broccoli)	Whole Wheat Pasta with *Turkey and Butternut Squash	Pork Tenderloin Roasted Apples Spinach and Farro Salad	Chicken Parmigiana Black Bean Brownie	Spinach Calzone Sweet Potato Fries	Salmon Cakes with Romesco Sauce (tomato, almonds, olive oil, green peppers, garlic) Roasted Potatoes Sesame Ginger Broccoli
Snacks	Orange Pear	Apple	Roasted Almonds Apple	Strawberry, Banana Chia Seed Smoothie	Walnuts Orange	Roasted Garlic Hummus Whole Wheat Pita Chips	Pear Apple

*All turkey sausages, turkey burgers, turkey patties, and turkey meatballs are made in-house, uncured/not processed

### Compliance, adherence, and adverse effects

Participants have regular counseling appointments with the RD via email and phone to support the patient in adhering to the provided diet and to make modifications, as necessary. While on the diet, participants are provided with food logs to record the estimated portion of food consumed and a description of any additional food eaten.

The study coordinator remains in regular contact with participants to track study procedures and record adverse events (AEs). The investigator assesses and grades adverse events according to the Common Terminology Criteria for Adverse Events (CTCAE) Version 4.0. Each AE can be attributed to the diet intervention, the ICB, or a combination of both.

### End of intervention

Participants in the unresectable and adjuvant cohorts remain on the provided diet for 10 weeks. The participants in the neoadjuvant cohort remain on the diet until the day of surgery for up to 10 weeks.

### Study discontinuation

Participants may be removed from the study if they experience any Grade III or above AE attributed to the diet intervention, if their treating oncologist requests removal, or if the investigator determines that they have a medical condition that may jeopardize their safety if they continue in the study. Participants can withdraw from the study at any time during the screening or over the course of the study. Reasons for withdrawal are recorded (e.g., inability to adhere to the intervention diet).

Participants who are removed or withdraw from the intervention can choose to stay in follow-up until the end of study.

### End of study and post-trial follow-up

To explore the post-trial impact of a change in diet, follow-up is conducted 12 weeks after the end of the dietary intervention. The participants are asked to complete diet and lifestyle surveys. Stool and optional blood samples are collected.

## Biospecimen and data collection

### Stool, blood, and tissue samples

The Participants are provided a schedule of stool collections at the start of the study. An in-home, fresh-frozen stool sample collection kit, similar to that used in the Human Microbiome Project (HMP) [24] and refined in our previous studies, is provided to participants with detailed instructions during the clinic visit. Participants can provide a same day fresh collection at MDACC or a fresh-frozen collection at home and return it via pre-paid mail with appropriate icepacks to maintain a temperature of 4–6 °C. Upon receipt, the samples are immediately processed and transferred to -80 ^o^C for storage.

Blood (30 ml) is collected at screening (optional), before each infusion cycle during the intervention if the participant is receiving infusions at an MDACC Houston area location, at the end of the intervention, and at the end of the study. Up to 50 ml of blood is collected for peripheral blood mononuclear cell (PBMC) isolation, plasma and serum and cryopreserved for subsequent evaluation.

All participants in the neoadjuvant arm are required to have tumor samples collected before the intervention and at the time of surgery. Participants must have archival tissue available or be willing to undergo core biopsy or incisional procedures at baseline. Tumor tissue collection is optional for participants in unresectable cohorts. The tissue is collected according to the MDA guidelines and standard operating procedures. All samples are processed, labeled, and stored in accordance with NCI best practices for biospecimens.

### Dietary compliance

At baseline and at the end of the study, patients complete multiple dietary records and the Dietary History Questionnaire (NCI-DHQ) to determine the usual composition of their diet, including total energy intake (kcal), macronutrient composition (e.g., percent energy from fat), dietary fiber intake and overall diet quality [29]. 

Compliance is defined as the proportion (%) of calories consumed versus provided. The participants’ diets are continually assessed and monitored for compliance throughout the study via daily food logs reviewed during weekly appointments with the RD. Participants are considered adherent if ≥ 70% of weekly calories consumed by the participant are from the provided diet over the duration of the study.

### Lifestyle assessment

Quality of life is assessed using the European Organization for the Research and Treatment of Cancer Quality of Life Questionnaire Core 30 (EORTC QLQ-C30), a 30-question assessment of quality of life in cancer patients [30]. Gastrointestinal symptoms are assessed via the Gastrointestinal Symptom Rating Scale developed for patients with irritable bowel syndrome (GSRS-IBS) [31]. Physical activity levels are assessed via a validated long version of the IPAQ at screening and at the end of treatment [32]. 

Fitbit devices are provided to monitor physical activity throughout the duration of the study.

### Data quality and integrity

All the data are stored in a password-protected secure web-based application, Research Electronic Data Capture (REDCap) and shared folders on a secure and routinely backed-up institutional server.

## Laboratory methods

### The gut microbiome and metabolome

The goal of the current study is to establish the effects of dietary intervention on the structure and function of the gut microbiome in patients with melanoma treated with SOC ICB. The gut microbiome composition will be characterized using whole-genome shotgun (WGS) sequencing of the collected stool samples. Mass spectrometry-based metabolomic profiling will be performed on serum and stool samples collected from the participants at baseline and throughout the intervention.

### Microbiome sequencing and data processing

Specimens from baseline to the end of the intervention will be compared longitudinally. Genomic datasets define the microbial composition of the microbiome at a given time point. Genomic sequence read datasets will be analyzed to assign a taxonomic identity at the resolution of an operational taxonomic unit (OTU) and to define the relative proportion of each OTU to all other OTUs in a given sample. Changes in the composition of the gut microbiome will be measured in terms of the total number of unique types of bacteria (i.e., α diversity) and the microbial composition (i.e., β diversity). Nonparametric and machine learning computational methods will be further applied.

### Fecal and serum marker analysis

In fecal and serum samples, circulating SCFAs, bile acids and global metabolites are measured using LC-MS/MS by Metabolon. Inc. Metabolites associated with host-, nutrient- or microbial-driven signaling will be analyzed over time.

### Immune profiling

Tumor tissue from the neoadjuvant and unresectable (if available) cohorts collected at baseline and at the end of the intervention will be collected. NanoString GeoMx Digital Spatial Profiler (GeoMx DSP) will be used to evaluate the immune environment using regions of interest (ROI)-guided selection and a gene expression panel (Whole Transcriptome Atlas (WTA) Panel; 18,000 + protein-coding genes). Plasma cytokines will be quantified via the O-Link platform.

## Statistical considerations

### Power

The sample size justification was based on the estimation of the mean difference between two treatment (diet) arms with respect to the change in the abundance of microbiome taxa from baseline to the end of the intervention (e.g., Clostridiales and Ruminococcaceae). Given 30 patients in the intervention group (i.e., high-fiber diet) and 15 patients in the control diet group, a 95% confidence interval for this mean difference would have a half-width = 0.62*s, where “s” is the common standard deviation of the changes. The study is not intended or powered for hypothesis testing, including comparisons between study intervention groups. This study is intended to provide preliminary estimates of effect sizes of changes in the microbiome and AE rates to aid in the design of future studies.

### Data analysis

For microbiome data analysis, alpha diversity, beta diversity, and abundance/relative abundance of different taxon levels will be compared between two arms using two-sample t tests or Mann‒Whitney tests. The linear discriminant analysis effect size (LEfSe) tool will be used to analyze and visualize relative abundance. The longitudinal trajectories of the alpha diversity and abundance/relative abundance of different taxa will be compared between treatment arms via linear mixed effects models with time‒treatment interactions. The assumptions will be verified using model diagnostics, and if not met, appropriate data transformations will be used. Demographic and clinical variables may be adjusted in the analysis as appropriate. The similarity in microbial community structure between treatment arms will be assessed and visualized by principal coordinate analysis (PCoA) of distance matrices such as unweighted and weighted UniFrac measures and compared using the permutation-based nonparametric MANOVA. We will use the intention-to-treat population for the primary analysis. We will use the last observation carried forward method to impute missing data. If a patient drops out before the end of the intervention, the last observed microbiome profile values will be used for all subsequent observation points.

We will also explore associations between baseline patient characteristics with microbiome profiles (e.g., alpha diversity and relative abundance), immunity, metabolism (e.g., gene expression) and quality of life at baseline and at the end of intervention. Pearson’s or Spearman’s correlation coefficient will be used to assess the correlation between measures for the microbiome profile, immunity, metabolism and quality of life at baseline and at the end of the intervention.

The ORR in the unresectable cohort and the RR in the adjuvant cohort will be estimated along with the 95% confidence interval, and comparisons between the two diet groups will be assessed using chi-square test or Fisher’s exact test, as appropriate. The PFS rate in the unresectable cohort and comparisons between the two diet groups will be evaluated via the log-rank test.

Safety and tolerability will be assessed through summaries of AEs attributed to diet as well as irAEs attributed to immunotherapy and gastrointestinal symptoms (GSRS-IBS) using frequency counts and percentages. Other statistical approaches might be employed.

## Discussion

Controlled feeding studies are a gold standard in human nutrition research to minimize underlying variations in eating behaviors that impact findings in other dietary intervention settings [33]. Paralleling to preclinical studies, these designs allow for proof-of-principal testing of the impact of a specific diet on biology.

To our knowledge, this is the first ever controlled feeding study in active cancer patients treated with immunotherapy. It also exceeds the length of many prior controlled feeding studies in other settings, with rich biological correlates. Current dietary guidelines for patients with cancer are largely extrapolated from the cancer prevention literature or focused on acute issues during treatment. Notably, they are not specific to histology, stage, or treatment type and can be used to inform nutritional strategies in therapeutic settings. HFDIs are typically well tolerated; however, there is the potential for diarrhea, gas, and bloating, which could overlap with ICB-induced colitis; hence, the safety and tolerability of this combination need to be tested. Both ACS and AICR have released similar dietary recommendations for cancer prevention [23, 24], including a diet rich in whole grains, vegetables, fruits and beans with limited red and processed meat; sugar-sweetened beverages; alcohol; and other processed foods. For patients with a diagnosis of cancer, these organizations suggest that the above dietary guidelines are followed “if you can” and there is data supporting that a “prudent” diet may positively impact long-term survival outcomes [34]. However, the effect of this diet + ICB on the antitumor immune response has not been well studied.

Resistant starches and dietary fiber are prebiotics that are indigestible by the host and fermented and metabolized by bacteria that produce short-chain fatty acids (SCFAs), which play a key role in maintaining intestinal homeostasis and promoting high microbial diversity and low pathogen abundance. SCFAs and other bacterial metabolites appear to play a key role in microbiome-immune interaction by affecting the intestinal epithelial barrier and defense functions, regulating innate immune cells and the differentiation and activation of T cells and B cells [19, 35–38]. More recent work has suggested that SCFAs can also directly impact cytolytic T-cell activity, with microbiota-derived SCFAs enhancing the memory potential of activated T cells via epigenetic regulation [39]. A prior controlled feeding study utilizing a 50 g of fiber/day intervention for 2 weeks demonstrated rapid and reproducible increases in SCFA-producing bacteria [38]. These authors further demonstrated changes in gut mucosal biomarkers of cancer risk, supporting that dramatic shifts in diet can have equally dramatic effects on the microbiome and cancer biomarkers. However, the diet-microbiome-metabolite-immunity axis has not been studied to date in the context of immunotherapy. The biospecimen analyses of the current study will provide a deep understanding of high fiber diet induced changes in metabolomics and immunity and further potentially improve predictors of outcomes and strategies to thwart melanoma recurrence and therapeutic resistance.

## Data Availability

No datasets were generated or analysed during the current study.

## Abbreviations

DIET: Diet and Immune Effects Trial
ICB: immune checkpoint blockade
HFDI: high-fiber dietary intervention
SOC: standard of care
PD-1: programmed cell death protein 1.
CTLA-4: cytotoxic T-lymphocyte associated protein 4.
LAG-3: lymphocyte activation gene-3.
FMT: Fecal microbiota transplantation
GI: gastrointestinal
ORR: objective response rate
PFS: progression-free survival
RR: recurrence rate
ACS: American Cancer Society
AICR: American Institute for Cancer Research
BRC: Bionutrition Research Core
MDACC: MD Anderson Cancer Center
IRB: Institutional Review Board
RD: registered Dietitian
IPAQ: International Physical Activity Questionnaire
CTCAE: Common Terminology Criteria for Adverse Events
AEs: adverse events
HMP: Human Microbiome Project
PBMC: peripheral blood mononuclear cell
DHQ: Diet History Questionnaire
EORTC QLQ-C30: European Organization for the Research and Treatment of Cancer Quality of Life Questionnaire Core 30
GSRS-IBS: Gastrointestinal Symptom Rating Scale developed for patients with irritable bowel syndrome
WGS: whole-genome shotgun
OTU: operational taxonomic unit
ROI: regions of interest
WTA: Whole Transcriptome Atlas
PCoA: principal coordinate analysis
SCFAs: short-chain fatty acids
